# Organoid as a promising tool for primary liver cancer research: a comprehensive review

**DOI:** 10.1186/s13578-024-01287-5

**Published:** 2024-08-27

**Authors:** Xuekai Hu, Jiayun Wei, Pinyan Liu, Qiuxia Zheng, Yue Zhang, Qichen Zhang, Jia Yao, Jingman Ni

**Affiliations:** 1 School of Pharmacy, Lanzhou University, Lanzhou, 730000 China; 2 The First school of Clinical Medicine, Lanzhou University, Lanzhou, 730000 China; 3 The First Hospital of Lanzhou University, Lanzhou, 730000 China; 4 Key Laboratory of Biotherapy and Regenerative Medicine, First Hospital of Lanzhou University, Lanzhou, 730000 China; 5 School of Basic Medical Sciences, Key Laboratory of Preclinical Study for New Drugs of Gansu Province, Lanzhou University, Lanzhou, China; 6 The First Hospital of Lanzhou University, No. 1 West Donggang Road, Lanzhou, Gansu 730000 P. R. China; 7 School of Pharmacy, Lanzhou University, No. 199 West Donggang Road, Lanzhou, Gansu 730000 P. R. China

**Keywords:** Primary Liver Cancer, Organoid, Tumor Research, Drug screening, Personalized medicine, Translational research

## Abstract

Primary liver cancer (PLC) is one of the most common malignant gastrointestinal tumors worldwide. Limited by the shortage of liver transplantation donors and the heterogeneity of tumors, patients with liver cancer lack effective treatment options, which leads to rapid progression and metastasis. Currently, preclinical models of PLC fall short of clinical reality and are limited in their response to disease progression and the effectiveness of drug therapy. Organoids are in vitro three-dimensional cultured preclinical models with a high degree of heterogeneity that preserve the histomorphological and genomic features of primary tumors. Liver cancer organoids have been widely used for drug screening, new target discovery, and precision medicine; thus representing a promising tool to study PLC. Here, we summarize the progress of research on liver cancer organoids and their potential application as disease models. This review provides a comprehensive introduction to this emerging technology and offers new ideas for researchers to explore in the field of precision medicine.

## Background

Primary liver cancer (PLC) is the sixth most common malignant tumor and the third leading cause of cancer-related deaths in humans [1]. With high recurrence and metastasis rates, low detection rates in curable stages, and ineffective treatment options, PLC is associated with poor prognosis. Based on the main pathological type, PLC can be classified as hepatocellular carcinoma (HCC, 75–85% of cases), intrahepatic cholangiocarcinoma (ICC, 10–15% of cases), and combined hepatocellular carcinoma-intrahepatic cholangiocarcinoma (cHCC-ICC). Different degrees of PLC present with different cell types and levels of differentiation, resulting in high tumor heterogeneity [2]. The hepatic parenchyma consists mainly of two cell populations, hepatocytes and biliary cells, which remain highly plastic during injury and regeneration. When proliferation is impaired on one side, an alternative regenerative mechanism occurs on the other side. This is demonstrated by the phenomenon that hepatocytes can temporarily or permanently trans-differentiate into the biliary phenotype, and biliary cells can stably differentiate into hepatocytes [3]. Several factors contribute to PLC, with viral infections being prominent risk factors. Hepatitis B virus (HBV) and hepatitis C virus (HCV) play a crucial role in mediating tumorigenesis and transformation, with viral carriers being a well-defined category of high-risk individuals. The remaining influencing factors involve altered hepatocyte metabolism, which includes obesity, nonalcoholic fatty liver disease, metabolism-related fatty liver disease, type II diabetes and drug toxicity overexposure, all of which damage hepatocytes. These damaged hepatocytes subsequently undergo inflammation, leading to liver fibrosis, cirrhosis, and end-stage liver disease [4, 5].

PLC is a multistep process, accompanied by associated gene mutations and dysregulation of signaling pathways; resulting in inter and intra-tumor heterogeneity. The optimal treatment requires multidisciplinary participation and the coexistence of multiple therapeutic modalities. The choice of treatment regimen must consider the patient’s systemic status, typical manifestations of the tumor, liver function, and the response to treatment. Treatment routes and prognostic assessments are mainly based on the staging of liver cancer. Several staging protocols and scoring systems such as the Barcelona Liver Cancer Staging system, China Liver Cancer Staging Programme, Tumor Node Metastasis staging system, and bilirubin, albumin (ALB), Lens culinaris agglutinin A–reactive fraction of alpha-fetoprotein (AFP), and des-γ-carboxy prothrombin score are in use [6]. Treatment strategies change with disease progression. For example, in the early stage of HCC, surgical treatment and ablation therapy are often used, with surgical treatment achieving radical resection of HCC for long-term survival and ablation therapy providing a less invasive solution with precise efficacy. Various physical and chemical ablation techniques have been developed to treat multiple tumors and metastases. Transcatheter arterial chemoembolization (TACE) and radiotherapy have been used for intermediate-stage HCC. TACE kills tumor cells locally by blocking the hepatic or extrahepatic arterial collateral blood supply and involves the injection of chemotherapeutic drugs, whereas radiotherapy uses high-energy rays and radionuclides implanted in body tubes. Other systemic antitumor treatment options such as chemotherapy, molecular targeted therapy, immunotherapy, and gene therapy can also be used for terminal HCC [7]. These treatment options must consider the temporal (disease process) and spatial (disease site) differences. Therefore, preclinical models of liver cancer have been developed to refine and improve treatment options as well as achieve precise patient treatment.

Preclinical trial modeling is a key approach in medical research as it provides preliminary information on new drugs or treatments and ultimately helps investigators assess their safety, activity, and pharmacokinetic properties. Preclinical models of PLC are divided into in vitro and in vivo models. In vitro models include two-dimensional (2D) monolayer cell cultures, co-cultures, organoids, and three-dimensional (3D) printed models, whereas in vivo models include chemically induced, patient-derived xenograft (PDX), and genetically engineered mouse, rat, and zebrafish models [8, 9]. Among these, organoids, as an emerging cell culture technology, have improved the limitations of existing models and demonstrated strong application prospects. Organoids are complex cellular aggregates formed by different types of cells (stem, progenitor, and differentiated cells) cultured in vitro that exhibit a 3D multicellular structure with the ability to self-assemble and differentiate [10]. Organoids facilitate cell-cell and cell-cell matrix interactions and secrete factors that drive cells to undergo proliferation, differentiation, sorting, self-organization, lineage commitment, and morphogenesis [11]. Organs from which organoids have been established so far include brain [12], retina [13], heart [14], liver [15], and pancreas [16]. In addition to the establishment of organoid systems for normal tissues, tumor organoids have been developed as individualized cancer models by optimizing culture protocols for thyroid [17], lung [18], liver [19], ovary [20], and colon cancer [21].

In this review, we introduce the current sources and methodologies for establishing liver cancer organoids and describe the application of organoids from the perspectives of basic research, drug discovery, and clinical studies. We also discuss the limitations of organoid technology and provide an outlook for the future development of liver cancer organoids.

### Establishment of liver cancer organoids

#### Organoid culture

Organoid culture involves tissue extraction or stem cell culture, followed by the application of suspension culture, rotational biomass, gas-liquid interface, and extracellular matrix (ECM) scaffolds for generation [22]. Organoid culture systems mainly contain soluble signaling molecules, ECM, culture media, and supplements [23].

In organoid culture, soluble components are presented as biological agents, primarily comprise growth factors, small molecule drugs, hormones, and cellular metabolites. These components can activate or inhibit signaling pathways, thereby regulating the development and function of organoids. For instance, by collecting the supernatant of the culture medium from engineered cell lines L-Wnt-3 A and 293T-HA-Rspo1-Fc, a conditioned culture medium, which affects Wnt signaling pathway can be produced [24, 25]. Noggin, as an inhibitor of bone morphogenetic protein 4 and bone morphogenetic protein 7, can inhibit the differentiation of stem cells. The current study found that most liver cancer organoids derived from patients can grow in the absence of Wnt3a, Noggin, and/or R-spondin conditioned medium. However, for normal liver organoids, these bioactive factors are still necessary [26]. In addition, there is an effective distribution of soluble components in terms of time, space, and dosage. Soluble factors vary significantly in different tissues in terms of content and functional activity. The use of each soluble component may vary according to the specific purpose of the study and the type of tissue sample. These multivariate factors have been used since the authors are describing different type of factors to construct the occurrence of complex tissue patterns, such as mediating immune regulation and tissue regeneration [27, 28].

The ECM consists of water, proteins, polysaccharides, and structural molecules that mimic the microenvironment around an organ and provide structural support for organoids [29]. Cells sense changes in matrix geometry, mechanical properties, stress relaxation rates, and cross-linking rearrangements and respond influencing cell differentiation, proliferation, migration and angiogenesis [30]. Matrigel or basement membrane extracts (BMEs) are widely used as traditional culture matrices, which are considered the gold standard for in vitro cell culture [31]. However, Matrigel and BMEs are associated with poorly defined composition and high batch-to-batch variability, resulting in uncontrolled shape and size during organoid development [32]. Through continuous efforts, researchers have gradually discovered alternatives to matrix gel-derived materials, such as biopolymers (protein matrices such as collagen, fibronectin, and laminin and polysaccharide matrices such as alginate and hyaluronic acid), fully synthetic materials (polyethene glycol, polyglycolic acid, and multivacancy polylactic acid), and bioheterotropic materials (matrices crosslinked with proteins, Arg-Gly-Asp peptides, and natural polymers) [33, 34]. These engineered materials have been used for tumor organoid culture as well as the generation of vascular epithelium and osteogenic tissue.

The basal medium includes saccharides, nucleotides, and vitamins that meet the basic needs of cells and tissues and are involved in cellular energy metabolism and differentiation [35]. Supplementation often promotes neuronal differentiation and embryonic growth [36].

Briefly, the culture protocol for tumor organoids involves the digestion of tissues into cell clusters, inoculation of cell suspensions premixed with biomaterials in well plates, and subsequent supplementation with expanded media components. Core media components include N-acetylcysteine, nicotinamide, N2 supplementation, B27 supplementation, epidermal growth factor (EGF), hepatocyte growth factor (HGF), and fibroblast growth factor (FGF) [19]. N-acetylcysteine acts by modulating the phosphatidylinositol-3 kinase/protein kinase B(AKT) signaling pathway, and nicotinamide inhibits cell differentiation to promote organoid growth. Both serum-free supplements increase the viability of differentiated cells over a prolonged period [37]. EGF, FGF, and HGF are essential growth factors for hepatocytes. EGF acts as a signaling molecule that affects cell proliferation and survival. FGF acts on epithelial and endothelial cells by stimulating mitosis, tumor cell migration, and invasion while inducing the differentiation of hepatic progenitors into hepatocytes to some extent. HGF can bind to heparin, affecting tissue remodeling [38]. Under the influence of EGF, FGF, and HGF, hepatocytes can proliferate rapidly and remain active for long periods of time.

In addition to these essential components, organoid culture protocols vary among laboratories. For example, gastrin, dexamethasone, and tumor suppressor M can be added selectively [15, 39–41].

#### Organoid purification

Because tumor tissue samples also contain contamination by normal cells, the tumor organoids obtained by culture may be mixed with normal organoids. Overgrowth of normal organoids from liver cancer samples and from other types of tumor tissues has been observed so far [19, 26, 42–44]. Normal tissue organoids are mainly cystic, with a few appearing as solid structures. In contrast, tumor organoids resemble their original tumors with varying morphologies and predominantly irregular structures. They exhibit monolayered, cystic, highly disorganized solid and grape-like parenchymal structures. This mixed culture will influence the cell biology research of tumors, leading to inaccuracies in experimental results. Therefore, it is necessary to establish purified tumor organoids.

Purification methods can be based on primary tissues or organoids. For primary tissues, flow sorting or magnetic bead sorting can be performed before organoid culture to improve the purity of tumor cells, and subsequently perform tumor organoid culture. Alternatively, the digestion time can be increased to avoid the growth of non-cancerous components in tumor organoids [19]. At the level of organoids, purification methods include phenotype-based manual selection of normal cell-derived organoids, sorting of organoids by fluorescence-activated cell sorting or by flow cytometry after digestion, and selection of culture medium more conducive to the growth of tumor organoids [45]. First, using the morphological and structural differences of organoids, normal organoids can be manually removed under the microscope. Secondly, dissociation of single organoids and subsequent cell sorting by flow cytometry can effectively obtain single cells of tumor organoids, thus establishing clonal cancer organoids. The third method selectively supports the growth of tumor organoids by adjusting the organoid culture medium. As mentioned earlier, medium without Wnt3a, R-spondin, and noggin, but supplemented with dexamethasone and Rho kinase inhibitor can inhibit the growth of normal liver organoids [46].

Finally, genetic analysis or immunohistochemistry has also be used to evaluate the purity of tumor organoids. For example, whole genome and whole exome sequencing can obtain the mutational profile of alleles and can further define the degree of contamination of organoids. Karyotype analysis of organoids, tumor organoids can often appear aneuploidy and present chromosomal instability [45, 47].

#### Organoid passaging and long-term preservation

Organoids contain a variety of cell types of the simulated organ, and grow by self-organization through cell sorting and spatially restricted lineage commitment [48]. At the macro level, the volume and number of organoids increase over time. In long-term culture, tumor organoids show no change in growth behavior or in morphological phenotype, allowing unlimited growth potential [24]. However, in vitro culture of organoids cannot form a vascular network, and nutrient supply and material transport are limited, which restricts volume growth and leads to insufficient function of organoids. At this time, it needs to be passaged for maintenance. The passage time and proportion of each established organoid should be optimized based on the growth confluence. At the time of passaging, organoids need to be recovered from the Matrigel and reduced in size by repeated mechanical blowing or chemical digestion. Subsequently, the single cells or multicellular fragments obtained are resuspended in Matrigel, supplemented with culture medium, and expanded into new organoids [49]. Alternatively, when significant apoptosis is observed in the organoid (such as shrinkage of the organoid or darkening of its appearance), necrotic or functionally impaired cells need to be removed by passaging to maintain the function of the organoids. After organoids are sheared into single cells or multicellular fragments, they can better receive nutrients and oxygen to avoid necrosis. After passaging, stem cells and other cells in organoids will continue to proliferate and will again undergo self-organization, thus generating new organoids.

Organoids can be stored in liquid nitrogen for long-term storage, and the specific steps include the removal of the matrix gel, centrifugal enrichment, and the addition of freezing medium [50]. After gradient cooling, organoids are transferred to liquid nitrogen for long-term storage. Even after freezing for several years, cell viability is not compromised. To obtain a high recovery success rate, small organoids in the exponential growth phase are usually frozen [25]. Organoids can be passaged and frozen, thus providing the possibility to establish a biological sample bank with high clinical relevance. Living organoid biobank is helpful for basic or applied research, and is expected to be shared globally [51]. Organoids in biobanks maintain their original characteristics after long-term culture and become valuable resources for subsequent research. Biobanks have been established for different cancer organoids, such as breast [52], stomach [53], colon [54], and lung [55]. Jin et al. developed the Liver Cancer Organoid Biobank (LICOB), which contains 65 patient-derived organoids. Using LICOB, researchers can explore drug response correlations from mutation, copy number variation, methylation, mRNA, protein, and pathway analyses using a web portal (http://cancerdiversity.asia/LICOB/) [56].

#### Characterization of organoids

**Fig. 1:**
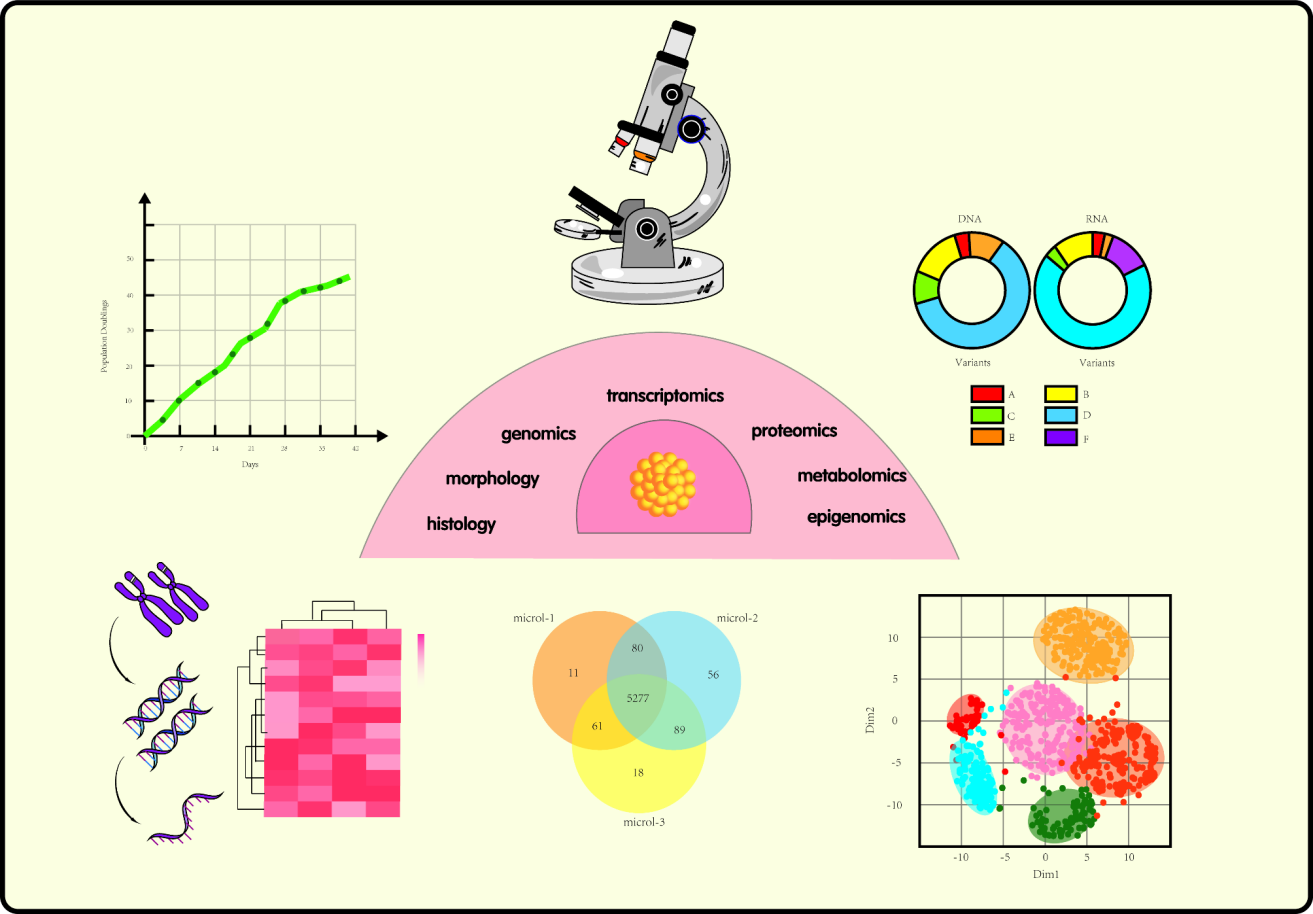
Techniques for characterizing organoids

Organoids recapitulate the histology and heterogeneity of the parental tissue. Histological diagnosis or multi-omics analysis of organoids is performed during their growth [57]. Organoids can be analyzed using microscopy or cell imaging to determine their number, size, and structure, whereas image processing software is further used to generate organoid growth curves (Fig. 1) [58]. Histological analysis can determine the source of organoids, observe tumor morphology, and determine the differentiation grade of cancer cells. Usually, after the embedding and permeabilization of organoids, hematoxylin-eosin (H&E) staining, immunohistochemistry, and immunofluorescence analyses are performed [59, 60]. The analysis of cell surface markers using flow cytometry also allows the characterization of organoids, as fluorescence-labelled antibodies and dyes can highlight the molecular targets on cells [61]. In addition to specific markers, flow cytometry can detect unique cellular functions, including cellular autophagy and epithelial-mesenchymal transition. Organoid function can also be assessed using cell viability. Cell viability measurement methods include ATP measurement (CellTiter-Glo), the use of cell-permeant dyes and tracers staining (Calcein AM green and MitoTracker Green), immunofluorescence staining based on replication markers (Ki67 and PCNA), and MTT/MTS/XTT measurement [62]. Finally, once organoids have been isolated, genomic and epigenomic characterization can be performed [63]. Tests related to the epigenome include whole-genome bisulfite sequencing to measure global DNA methylation levels, assays for transposase-accessible chromatin with high-throughput sequencing to assess chromatin accessibility, and chromatin immunoprecipitation technology to determine interactions between DNA and proteins. Conversely, genomics deals with conditions such as gene mutations, chromosomal instability, and somatic copy number abnormalities. The combined use of these techniques allows for a full range of comparisons between molecular subtypes and functional phenotypes, ensuring the successful establishment of organoid lines that are cell viable and retain the characteristics of the original tumor specimen.

## Liver cancer organoids from different sources

**Fig. 2:**
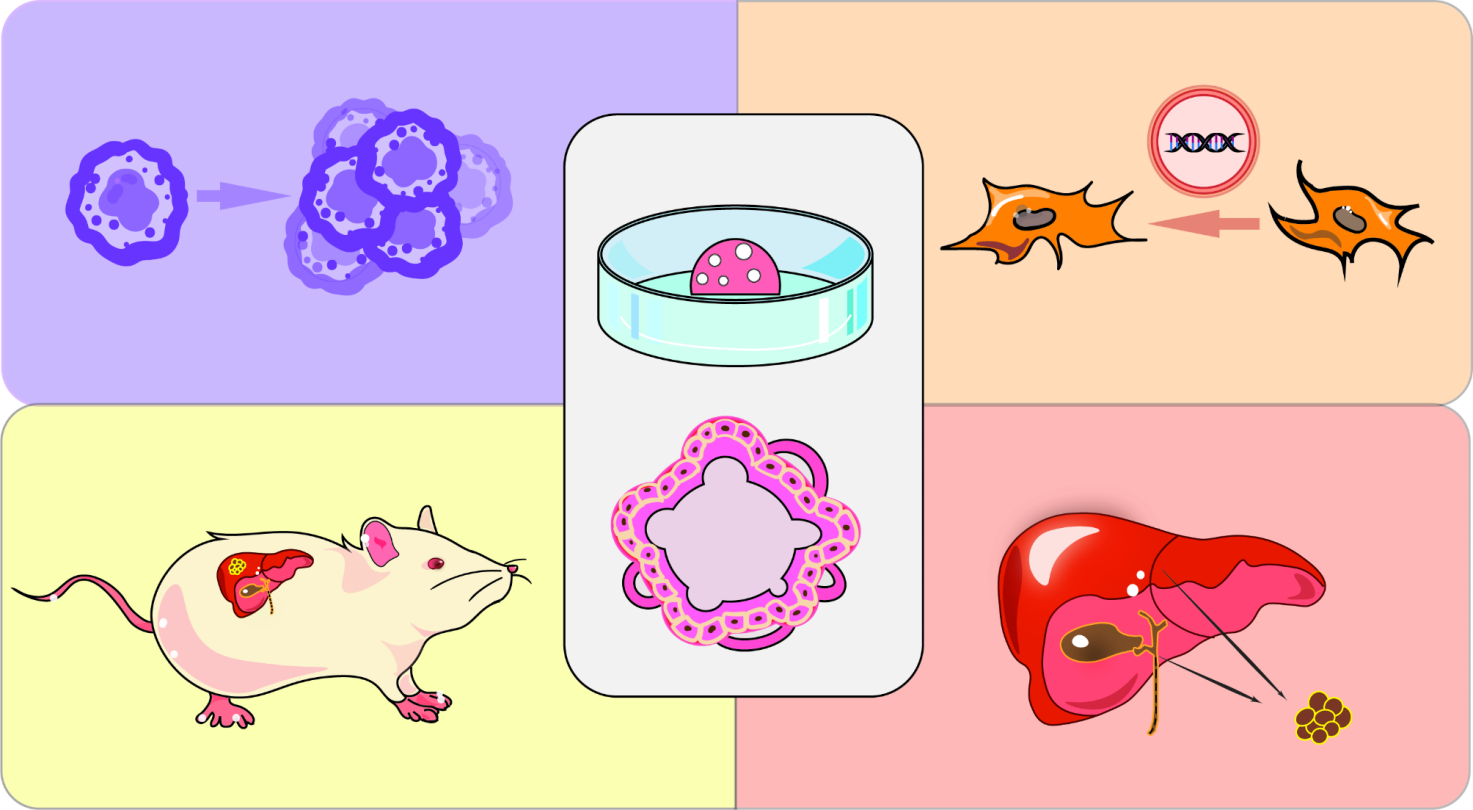
Different organoid models of liver cancer

Although stem cells and cancer cell lines are sources of liver cancer organoids, human and mouse tissues have been used to establish organoids (Fig. 2). Organoid models of liver cancer from different sources and their applications are summarized in Table 1. In 2009, Clevers et al. used Lgr5 intestinal stem cells to establish a culture system to maintain basic crypt-villus physiology in vitro. A single crypt can fission to produce multiple crypts, further forming a 3D structure with crypt-like and villous epithelial areas, that is, small intestinal organoids. This is the first truly self-renewing organoid that mimics the actual intestinal tissue structure. Since then, organoid research has entered a new era [64]. In 2013, Takebe et al. differentiated induced pluripotent stem cells (iPSCs) into hepatocyte-like cells and co-cultured them with umbilical vein endothelial cells and mesenchymal stem cells. These multiple type cells self-organized within 48 h to form macroscopically visible aggregates, known as “liver buds”, characterized by the formation of an endothelial network formation and expression of liver specific marker genes (AFP, ALB, retinol binding protein 4, transthyretin). After transplantation in mice, liver buds rapidly connected with recipient blood vessels to form a functional vascular network, resulting in a vascularized and functional mature “human liver”. This study provided a novel approach for the treatment of clinical transplantation of organs and has great prospects for regenerative medicine [65]. In the same year, Miho et al. used bioreactors to build a dense environment composed of fibrocytes and collagen fibers, and primary murine hepatocytes were encapsulated to form liver organoids comparable to endogenous liver. Morphological analysis revealed that hepatocytes in organoid tissues contained more organelles, such as mitochondria, Golgi apparatus, and endoplasmic reticulum, than those of hepatocytes in culture dishes. Moreover, structural liver organoid tissues exhibited a variety of liver specific functions, and microvascular networks could be formed in tissues after transplantation. Thus, organoids can be used to study the structural composition of an artificial liver [66]. Over time, advanced technologies, such as microfluidic platforms, biochips, and genetic engineering have been used for organoid establishment, and human as well as mouse liver cancer organoids have been used to provide diversity.

**Table 1:** Organoid models of liver cancer from different sources and their applications

Classification	Published Year	Cell/Tissue	Materials/Consumables	Application	Ref
Cell	2015	HEPG2, HCT116	HA hydrogel-coated microcarrier bead	Modeling tumor growth in vitro. Becoming metastatic tumor models. Drug screening. Understanding the effects of potential drug candidates on normal tissues surrounding tumors.	[71]
	2017	HCCLM3, Hep3B, HUVEC Human primary fibroblasts	Matrigel	Investigating the role of non-stromal cells in the cellular composition of HCC organoids. Increasing epithelial-mesenchymal transition and tumor malignancy. Increasing neovascularization.	[76]
	2018	WI38, LX2, HUVEC Primary hepatocellular carcinoma cells from HBV-infected patients: AMC-H1, AMC-H2	96-well round bottom ULA microplates	Comparing anticancer drug sensitivity in different culture methods. Optimizing the treatment of HCC.	[78]
	2021	iPSC-EC, iPSC-MC, Huh7-Luci	Ibidi culture-insert 2 well system	Generation of animal models of in situ cancer. Studying HCC tumor development under liver fibrosis TME.	[77]
Mouse liver tumor	2019	DEN-induced liver tumors	Matrigel	Initiating tumor formation in mouse xenografts. Drug evaluation. Promoting the development of targeted therapies for TIC.	[79]
	2023	*Tp53* and *Pten* double mutation-induced liver tumors	Matrigel	Assessing radiation sensitivity.	[80]
Patient-derived liver tumor	2017	Patient-derived PLC specimens	BME2	Constructing liver cancer organoids. Identifying potential prognostic biomarkers for primary liver cancer. Identifying patient-specific drug sensitivity. Searching for potential targets in primary liver cancer.	[19]
	2018	Tumor needle biopsies of liver cancers	BME2	Initiating tumor formation in mouse xenografts. Understanding tumor heterogeneity. Drug reaction. Biobank.	[40]
	2019	Patient-derived PLC specimens	Matrigel	Understanding intra-tumor heterogeneity. Understanding inter-patient drug response heterogeneity. Drug screening.	[41]
	2020	Patient-derived HB specimens	BME2	Modeling HB disease. Drug screening.	[86]
	2022	Patient-derived PLC specimens	Matrigel	Drug screening. Studying sorafenib resistance in HCC organoids. Exploring signaling pathways that inhibit HCC organoid growth.	[82]
	2022	Patient-derived FLC specimens	BME2	Modeling FLC disease. Drug screening.	[85]
Genetic engineering	2019	hiHep	24-well Kuraray ultra-low attachment plate	Simulation of liver cancer initiation. Performing lineage switching of hepatocytes. Introducing multiple mutations to mimic liver cancer progression.	[92]
	2023	Human fetal hepatocyte-like organoids	Matrigel	Modeling FLC disease. Investigating molecular pathogenesis.	[96]

BME2, basement membrane extract 2; DEN, diethylinitrosamine; EC, endothelial cell; FLC, fribolamellar carcinoma; HA, hyaluronic acid; HUVEC, human umbilical vein endothelial cell; HCC, hepatocellular carcinoma; HB, hepatoblastoma; iPSC, induced pluripotent stem cell; Luci, luciferase-expressing; MC, mesenchymal cell; PLC, primary liver cancer; TME, tumor microenvironment; TIC, tumor-initiating cell; ULA, ultra-low attachment

### Organoids derived from liver cancer cell lines

Human and animal cell lines are widely used for the in vitro modeling of liver cancer as they respond to specific genetic and epigenetic changes and are widely used for cell proliferation and metastasis studies. These lines include cells with hepatocyte-like (HepG2, HepRG, and Huh7) [67], cholangiocarcinoma cell-like (HuCCA-1 and MT-CHCO1) [68], and fibroblast-like (SKHep1 and SNU-475) phenotypes. Cancer cell lines are cost-effective, easily cultured, and infinitely transmissible. Single or multiple cancer cells can spontaneously aggregate in an ideal reactor and mix to form a spheroidal model with some of the characteristics of in vivo tumors, including a multilayered structure resembling tissues and the continuous multiplication dynamics of cancer cells [69]. Leite et al. generated a spheroidal structure of HepaRG through dimethyl sulfoxide-induced differentiation in a bioreactor culture for seven weeks. The core consisted of biliary cells, and the periphery consisted of hepatocytes. In addition to morphological and structural similarities, the organoids exhibited CYP450 enzyme activity and hepatotoxicity to drugs [70]. In another study, HepG2 hepatocellular carcinoma cells and HCT-116 human metastatic colon cancer cells were co-cultured, with hyaluronic acid and gelatin providing a customized spatial structure for these cells. The average size of the generated organoids steadily increased over time. The expression patterns of HCT-116 cell surface markers under different culture conditions were compared. In the 2D environment, cells were only observed with epithelial phenotype (zona occludens 1[ZO-1] and β-catenin), while mesenchymal markers (N-cadherin) were negative. When the cells transited to the organoid environment, they showed weak expression of ZO-1, E-cadherin, and vinculin. However, β-Catenin, N-cadherin, and matrix metalloproteinase-9 (MMP-9) were expressed. This suggests that the organoid environment supports a shift towards mesenchymal, mobile, and metastatic phenotypes. The authors of the study demonstrated that liver tumor hybrid organoids can provide a better metastatic tumor model compared to 2D cultures [71].

Although every tumor cell line can generate organoid structures, such culture systems do not contain a complete tumor microenvironment (TME). Cancer cell survival is dependent on the TME, as it regulates cell survival, renewal, and differentiation, and influences the gradient distribution of nutrients, metabolic waste, and drugs [72]. The TME is a highly complex structural system composed of cellular and non-cellular components. Various cells in the TME can be tumor suppressive or tumor-promoting. Including tumor cells, immune cells (adaptive immune cells, myeloid immune cells, innate immune cells), stromal cells (cancer-related fibroblasts, adipocytes, neurons, and nerves), and vascular cells (vascular endothelial cells, lymphatic endothelial cells, pericytes) [73]. Non-cellular components include ECM, growth factors, cytokines, chemokines, enzymes, matrix proteins, and metabolic intermediates, which perform the function of information exchange [74, 75]. Wang et al. used Matrigel to grow HCC cell lines (HCCLM3 and Hep3B) along with non-parenchymal cells under 3D conditions and efficiently self-organized them, ultimately forming macroscopically visible organoids within 24 h post-inoculation. The addition of endothelial cells and fibroblasts enhanced the expression of angiogenic markers (vascular endothelial growth factor receptor 2, vascular endothelial growth factor, hypoxia-inducible factors-α), tumor-associated inflammatory factors (C-X-C-motif receptor 4, C-X-C motif chemokine ligand 12, tumor necrosis factor-α), and molecules related to epithelial-mesenchymal transition (transforming growth factor-β, vimentin, MMP9) compared with single HCC organoids. This model was used to verify that non-parenchymal cells are important components in the formation of liver cancer organoids. It can also be used as a stable and easy-to-operate organoid generation method for high-throughput assays [76]. Huh7 is a commonly used HCC cell line. Qiu et al. generated complete HCC organoids using luciferase-expressing Huh7 cells, endothelial cells, and mesenchymal stromal cells derived from human iPSCs. Organoids expressed specific immunofluorescence signals of these three cells (AFP, hCD31, and vimentin). The formed organoids secreted ALB and exhibited liver functional activity. In addition, the authors also introduced different types of controllable TME into organoid models to study the role of the TME in HCC tumor growth. For example, HCC organoids were orthotopically implanted into the liver of mice with fibrosis, and it was found that a fibrotic TME could promote tumor proliferation unidirectionally, while tumor proliferation could not promote the level of fibrosis. However, the development of HCC tumor is not significant in the model of nonalcoholic fatty liver disease. Finally, the organoids were implanted into humanized mice, which resulted in smaller tumors. This indicates that the immune response is important for slowing down tumor growth at early stage. The authors also found that in addition to Huh7, other liver cancer cell lines, such as HepG2, could also successfully form liver cancer organoids. This orthotopic liver cancer model established by using tumor organoids is beneficial to study the TME and tumor development process of HCC [77]. In addition to using the existing cancer cell lines, Song et al. obtained cell lines named AMC-H1 and AMC-H2 from the primary tissues of two HBV infected HCC patients. These two cancer cells can achieve cohesion in a short time, express AFP and have HBV DNA, and retain highly specific genetic changes. Co-culture of AMC-H1 and AMC-H2 with three types of stromal cells (LX2, WI38, and human umbilical vein endothelial cell) under 3D culture conditions formed multicellular tumor spheroids. The multicellular spheroids showed a more compact and clear morphology and a selective response to the standard therapies for HCC, such as sorafenib, 5-fluorouracil, and cisplatin. Thus, this model can be used for screening of selected drugs providing information for clinical drug application [78].

### Mouse liver tumor-derived organoids

Cao et al. used dimethylammonium nitrite to induce PLC in mice and established a liver cancer organoid model. A variable number of tumors appeared in the liver of each mouse, such that 129 liver tissue/tumors were obtained from 53 mice. Organoid culture of PLC mouse tumors resulted in the successful establishment of 91 models, with a success rate of 70.5%. These tumor organoids were morphologically diverse and could be cultured, frozen, or revived for long periods of time. After homografting in immunodeficient mice, approximately 20% of the derived organoids could rapidly re-initiate tumors, suggesting that the tumor-derived organoids in these mice were self-renewing and highly tumorigenic. Moreover, the tumor organoids expressed specific markers for hepatocytes (AFP/hepatocyte nuclear factor 4α) and cholangiocytes (epithelial cell adhesion molecule [EpCAM]/cytokeratin 19). The xenograft tumors had the same histological features and expression profiles as the primary tissues. In addition, the evaluation of response to anticancer drugs could be carried out in tumor organoid. It shows that organoids can promote the development of liver cancer (stem cell) biology, drug development and personalized medicine [79].

Jeon et al. injected plasmid vectors containing tumor suppressor genes into the livers of adult C57BL/6 mice via hydrodynamic tail vein injection. Genetic modifications that produce double mutations in tumor protein 53 (Tp53) and phosphatase and tensin homolog (Pten) induced the formation of liver tumors within approximately four months. By correlating human target gene data, it was found that the survival rate of neurofibromin2 (Nf2) or tuberous sclerosis complex 2 (Tsc2) low expression group was lower. Furthermore, Nf2 or Tsc2 gene mutations accelerated tumor formation in a double mutant background of Tp53 and Pten. Organoids were prepared from liver tumors generated in mice. Pten + Tp53 mutant organoids showed cystic structures, while Tp53 + Pten + Nf2 mutant organoids showed dense morphology. After the organoids were irradiated with the specified dose of radiation, the organoids with mutations showed different sensitivity to radiation. This study helped clarify the underlying mechanisms of individual differences in radiosensitivity by testing the radiosensitivity of liver tumors in vitro [80].

### Patient-derived organoids

Liver cancer-patient-derived organoids are mainly derived from punch biopsy and surgically collected tumor specimens [40]. In addition, body fluids from cancer patients (pleural fluid or ascites) have been used to establish pancreatic, breast, and lung cancer organoids [81].

Broutier et al. were the first to construct patient-derived PLC organoids by establishing liver and pancreatic organoid protocols with self-renewal capacity in humans and adult mice in 2016 [26]. They extended the culture system for healthy hepatocytes to pathology one year later by designing a liver cancer isolation medium based on normal liver organoid medium, which induces liver tumor organoid growth. First, specimens of the three main subtypes were obtained by surgical resection. Three HCC, three ICC, and two cHCC-ICC organoids with different degrees of differentiation were established. Organoids created using this method retained the specific morphology, marker analyses, transcriptomic features, and gene mutations of the tumor tissue of origin. For example, HCC organoids highly expressed HCC (AFP and glypican-3) and hepatocyte markers (ALB, apolipoprotein E, and transthyretin), but downregulated typical ICC markers, which are some ductal-related markers. In contrast, the ICC organoids highly expressed ICC markers (EPCAM and S100 calcium binding protein A11). Injection of patient-derived organoids into the subcutis of immunodeficient mice revealed tumor growth, and the resulting tumors strongly resembled organoids. This finding suggests that PLC-derived organoids have the potential for tumor formation and metastasis after long-term in vitro expansion and in vivo transplantation. In addition, liver cancer organoids can identify patient-specific drug sensitivities. A correlation has also been shown between the sensitivity of some drugs and the mutational spectrum. Interestingly, the extracellular regulated protein kinases (ERK) inhibitor SCH772984 can inhibit the growth of some HCC and ICC tumor organoids. In vitro experiments also proved that SCH772984 could significantly reduce tumor growth, indicating that SCH772984 may represent a potential therapeutic agent for PLC. Tumor organoids can replicate the histological, transcriptomic, and genomic profiles of PLC in vitro, helping researchers in their deeper understanding of liver cancer biology and the development of personalized treatments [19].

In 2022, Xian et al. established 52 organoids from 153 patients with PLC, with a success rate of 29% for HCC organoids, 52.9% for ICC organoids, and 100% for cHCC-ICC organoids. Compared with the PDX model, the success rate of tumor organoid establishment was higher and took less time. The factors influencing organoid establishment were determined by comparatively analyzing the clinical data from patients: samples with larger tumor volumes, microvascular invasion, terminal PLC stage, and high levels of AFP expression had a greater likelihood of establishing successful organoids. The occurrence of sorafenib resistance represents a difficult obstacle in the treatment of HCC. Acquired sorafenib-resistant organoids were generated form four HCC patients with acquired resistance to the drug. It was found that the resistant organoids were enriched in genes related to cancer stemness-related gene (myelocytomatosis viral oncogene homolog [c-myc] and epidermal growth factor receptor-related gene) and epithelial–mesenchymal transition-related gene (transforming growth factor-β1 and elongation factor 2-related gene), but the heterogeneity between organoids was still obvious. Further testing revealed that targeting the mechanistic target of the rapamycin (mTOR) signaling pathway could effectively treat acquired sorafenib-resistant HCC, which may be caused by inducing phosphorylated S6 kinase. This study not only generated a wide range of PLC organoids but also acquired sorafenib-resistant HCC organoids. It will help clarify the mechanism of acquired drug resistance in HCC and screen possible targeted therapies for HCC [82].

Fibrolamellar carcinoma (FLC) of the liver is a rare subtype of HCC, comprising approximately 1% of HCC in all patients. FLC has no gender preference, and its distinctive feature is the unimodal age distribution between young teens and thirties. About 80% of FLC occurs in adolescents and young people without previous liver disease. Patients harbor a 400 kb deletion on chromosome 19, producing a fusion between exon 1 of DnaJ heat shock protein family (Hsp40) member B1 (DNAJB1), a heat shock protein, and exons 2–10 of protein kinase cAMP-activated catalytic subunit alpha (PRKACA), the catalytic subunit of protein kinase A [83]. In situ hybridization and/or reverse transcription polymerase chain reaction to detect DNAJB1-PRKACA fusion transcripts are very useful for the confirmatory of FLC [84]. Narayan et al. used tumor and adjacent non-tumor liver tissues from nine patients (mean age 22.6 years) to derive human liver organoids for FLC disease modeling. These organoids were divided into six normal organoids, three primary tumor organoids, and 12 metastatic organoids. Metastatic organoids originated from different anatomical sites including the liver, lungs, and omentum. *DNAJB1*-*PRKACA* fusion transcripts were detected by polymerase chain reaction in both the FLC tumor samples obtained and patient-derived organoids, and DNAJB1-PRKACA fusion proteins were detected using probes. H&E staining showed that the organoids were similar to their tumor of origin and had the polygonal morphology of FLC cells, as well as granular eosinophilic stroma and prominent nucleoli. Transcriptome analysis further validated the tight phenotype of the tissue of origin and the organoid; thus, suggesting that the organoid recapitulated the features of the primary tumor of the patient [85].

Moreover, hepatoblastoma, known as “childhood liver cancer”, organoids were also established. They can be used for modelling and drug testing of pediatric solid hepatocellular carcinoma [86].

In addition to resected liver cancer tissues, Nuciforo et al. discovered that organoids can originate from tumor needle biopsies. They collected samples from diagnostic puncture biopsies to obtain both tumor and non-tumor samples. HCC organoids showed dense globules without a lumen, and non-tumor tissues grew as unicellular layers of vesicles. Biopsy-based organoids retained the same pathological features, somatic genetic alterations, and mutational features as the original tumor. Transplantation into immunodeficient mice produced xenograft tumors [40]. In such situations, tumor needle biopsy allows for the simultaneous acquisition of multiple samples with little or no damage to the patient, thereby overcoming the limitations of surgically resected specimens. At the same time, this protocol enables the establishment of organoids before extended surgery, providing a reference basis for disease diagnosis and clinical medication [87].

### Genetically engineered organoids

Specific modifications of genomic sequences can cause changes in target proteins that control cell differentiation. Organoids can be designed to generate human liver cancer organoids by modifying genomic sequences, endowing the organoids with specific functions, and effectively modeling the disease [88]. Modifications are usually performed using both gene editing methods and targeted transport.

Using both viral and non-viral approaches to organoid modification, a target gene is specifically encapsulated and aggregated at the target site in response to the stable delivery of the gene into the organoid. When selecting vectors, the time of expression, cellular characteristics, and length of the genetic fragments must be considered. Viral approaches that stably deliver genetic information to the offspring by transfection often use adenoviruses [89], lentiviruses [90], or retroviruses [91]. Sun et al. pre-generated reprogrammed human hepatocytes (hiHeps) from fibroblasts and expanded them using simian virus 40 large T antigen. HiHep cells develop an eosinophilic cytoplasm and elliptical nucleus and are enriched in mitochondria, similar to mature hepatocytes. The formed organoids have the same structure and function as the liver. Later, lentiviral vectors for oncogene transfection were used to overexpress the c-myc oncogene and RAS profiles in hiHep organoids; thus, establishing oncogenic transformation models of HCC and ICC. The organoids formed after these gene modifications had the typical features of HCC and ICC cells and could even undergo hepatocyte-to-cholangiocyte lineage conversion [92].

The organoid model of the disease was constructed in vitro, and the disease-causing genes were precisely manipulated by gene editing technology. The two technologies complement each other and represent an approach that will significantly advance the clinical translation of organoids towards the development of personalized medicine [93]. Frequently used genetic methods include RNA interference, transposons, and CRISPR-Cas [94]. The CRISPR-Cas nuclease system achieves multiple purposes, including gene repair, mutation, knockout, knock-in, and fusion, based on its broad applicability, stability, and ease of use [95]. As mentioned earlier, FLC, as a special class of HCC phenotypes, can be reproduced using CRISPR technology in different genetic backgrounds, including protein kinase A-associated mutations and BRCA1 associated protein 1 (BAP1)-driven mutations. In this study, after the cloning products were generated, they were selected to establish clonogenic organoids. Although different mutational backgrounds have been shown to cause hepatocyte dedifferentiation, only the combined deletion of BAP1 and protein kinase cAMP-dependent type II regulatory subunit alpha led to hepatocyte trans-differentiation into hepatic ductal/progenitor-like cells. The engineered organoids showed a heterogeneous polycystic appearance with the loss of highly polarized tissue. In addition, FLC mutant organoid models and tumor tissues are generally similar, with altered gene and protein expression present in the original tumor being consistently altered in the organoid. Knockdown techniques can be applied to normal liver organoids, triggering the onset of HCC [96]. Genetic engineering can also be used to simulate the function of human cancer genes in normal liver organoids. For example, Lam et al. simulated the early stage of liver cancer by manipulating the TP53 status of knockout and overexpression of R249S in normal liver organoids. First, CRISPR knockout of TP53 in liver organoids produced in tumor-like morphological alterations, enhanced stemness, and unrestrained proliferation in vitro. Further, overexpression of mutant R249S in TP53 knockout organoids observes a spontaneous increase in tumorigenic potential and true HCC histology in xenografts. Mutation of TP53, rather than simple loss, confers early clonal advantages and pro-survival functions in hepatocarcinogenesis. It shows that the organoids after gene editing can facilitate the analysis of cancer mutations and provide an opportunity to monitor early stage tumorigenic changes [97]. Artegiani’s team used CRISPR/Cas9 technology to knockout BAP1 gene function in normal human cholangiocyte organoids. Compared with wild-type liver ductal organoids, BAP1 mutant organoids exhibited complete loss of cell organization and polarity, cells presented a very irregular shape, and increased motility. Transcriptomic analysis and quantitative mass spectrometry revealed changes in organoid junctions and cytoskeletal components after BAP1 mutation, such as claudin1, claudin2, and periplakin. Surprisingly, after restoring BAP1 expression in the nucleus, the morphology of organoids can be rapidly reversed and normal morphology can be reconstructed. It shows that organoids combined with genetic engineering can effectively reproduce the carcinogenic process of human tissues [98].

### Organoids in liver cancer research

**Fig. 3:**
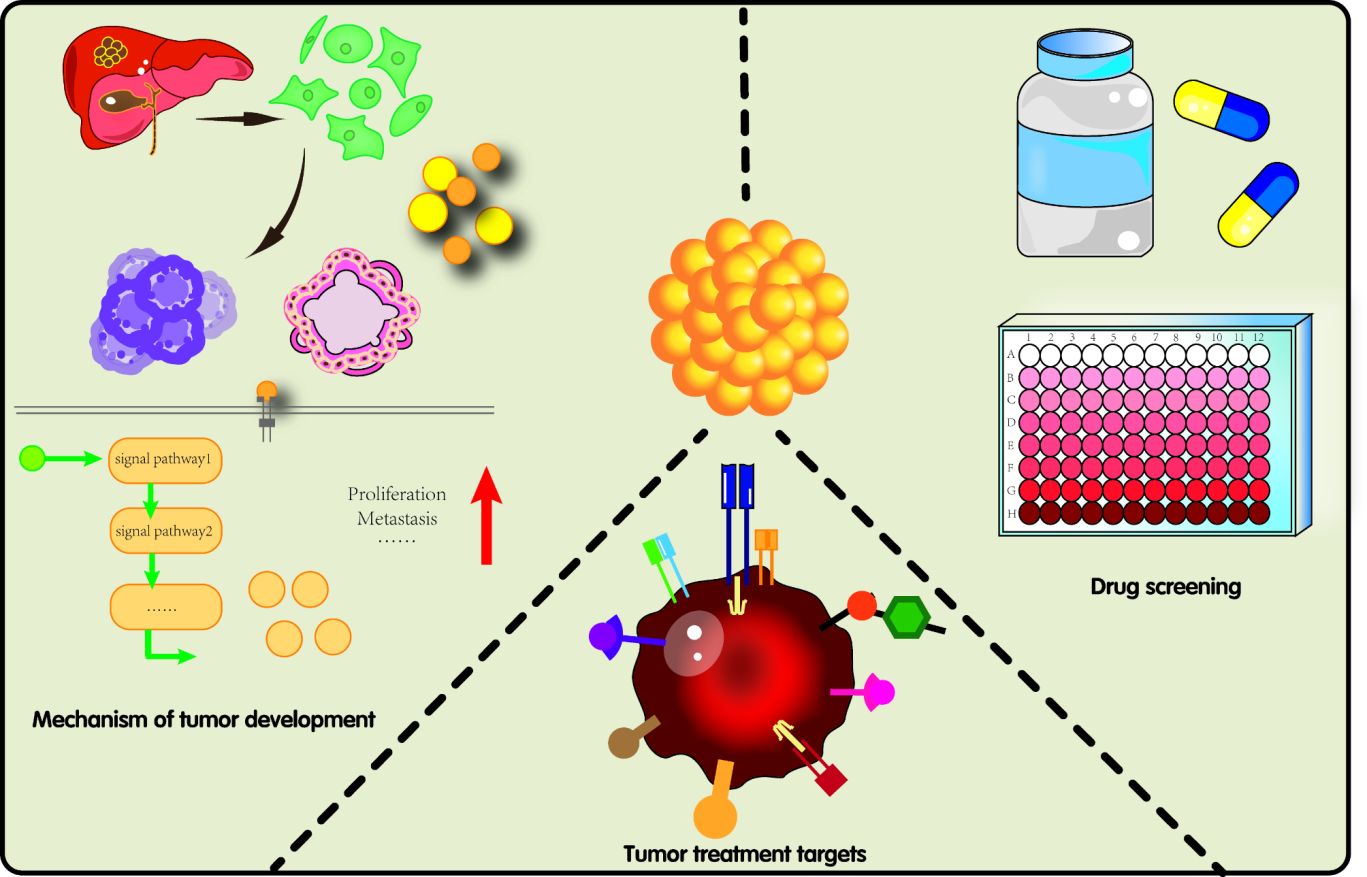
Organoid uses in liver cancer research

The main problems in the treatment of patients with cancer are the limited number of therapeutic modalities and wide variation in efficacy [99]. Organoids are promising in vitro models that preserve the genetic characteristics and molecular heterogeneity observed in patients. Compared with 2D cell culture models, the 3D structure of organoids provides a model that is closer to the physiological state, which can completely reproduce the system complexity and has patient-like heterogeneity. Compared with in vivo mouse models, organoids can be constructed at a lower cost, with a higher modeling success rate, which is conducive to short-term and large-scale preclinical screening of new drugs [100, 101]. Currently, organoids are widely used in tumor development, drug screening, regenerative medicine, and precision medicine, which can help elucidate the underlying mechanisms of PLC and promote its research development. Current uses of organoids in liver cancer research are illustrated in Fig. 3.

### Basic liver cancer research

#### Research on the mechanisms of tumor development

Simulating diseases at the tissue level is relatively difficult. However, organoids have a multicellular structure that corresponds to the state of cell development, proliferation, or regeneration under growth conditions. Several studies have reported the use of organoid models to explore tumor development, metastasis [102], and the TME [103]. Cancer-derived organoids retain tumor heterogeneity and recapitulate the parent tumor morphology, histopathology, hormone receptor status, and gene expression profiles [104–106]. At the same time, in more advanced biomedical research, the transformation and application of organoid technology can be accelerated by controlling the narrative engineering (morphogenesis, pattern, assembly, growth), biological environment (ECM, cell type, nutrients, stiffness), and comprehensive environment (gas, agitation, contraction, electrochemistry) in the process of organoid culture [27]. Furthermore, organoids allow the analysis of histological changes and mutated genes at the onset of cancer and the monitoring of liver cancer progression in vitro. For example, using organoids, activation of follistatin-like protein 1 (FSTL1) was shown to be correlated with the fibrotic state, whereas high FSTL1 expression was significantly associated with advanced liver tumors. By collecting conditioned media of hepatic stellate cell FSTL1 for the treatment of HCC cell lines and organoids, Loh et al. discovered that it promoted the growth and metastasis of HCC. If FSTL1 is blocked with the monoclonal antibody, the malignancy of HCC will be weakened. Similar results were found in HCC cell lines and organoids. In addition, western blotting confirmed that the key molecules in the AKT/mTOR/4EBP1/c-myc pathway (pAKT, p-mTOR, p-4EBP1, and c-myc) were enhanced in organoids treated with FSTL1; however, this pathway was inhibited after the addition of AKT inhibitor. These results indicate that FSTL1 drives hepatocarcinogenesis through the dysregulated AKT/mTOR/4EBP1/c-myc signaling pathway. It suggests that organoids can not only mimic the growth of HCC but they also allow to investigate molecular mechanisms leading to hepatocarcinogenesis [107].

Liver organoids are 3D cultures of bipotent liver precursors that can be manipulated to express cancer driver genes, which when are transplanted into mice can give rise to tumors. Cristinziano et al. used fibroblast growth factor receptor 2 (FGFR2) fusion proteins to design liver organoids from TP53 null mice. This liver organoid showed the characteristics of ICC after implantation in NOD-SCID mice. The tumors showed downregulation of genes linked to hepatocellular lineage, while genes of the transcriptional signature associated with embryonic cholangiocellular specification were upregulated. This suggests that FGFR2 fusion drives the transformation of bipotent liver organoids to cholangiocarcinomas. Furthermore, ERK1/2 was a necessary signal downstream of FGFR2 fusion in ICC. In this study, organoids and FGFR2 fusion proteins were used to understand biliary tumorigenesis and solve the problem of lack of available gene signature defined ICC models in current preclinical research [108].

Mimicking vascular secretion and the TME by co-culturing liver cancer organoids to identify the factors that drive tumor progression, including microbes, antigens, and inflammatory mediators, is possible. For example, the role of vascular secretory factors in regulating HCC progression was previously unclear. In addition to forming vascular structures, endothelial cells also coordinate cancer progression and metastasis through vascular factors. At present, many soluble and membrane-bound vascular secreted factors have been found, which can be roughly divided into endothelial cell adhesion protein (intercellular adhesion molecule 1, vascular cell adhesion molecule 1, E-selectin) and chemokines (interleukin-8 [IL-8], monocyte chemoattractant protein 1 [MCP1]). The patient derived organoids and endothelial cells was co-cultured in thiohyaluronic acid hydrogel, and the angiogenesis related proteins were analyzed using proteome. The secretion of MCP-1 and C-X-C motif chemokine ligand 16 was higher than that of PDX only or endothelial monocultures. The interaction between HCC and endothelial vessels also affects the immune microenvironment of HCC organoids, leading to the upregulation of vascular secreted factors (such as MCP-1 and IL-8) in co-cultures, promoting angiogenesis and macrophage polarization. Using organoids to investigate the effects of vascular secretory factors on the HCC phenotype and microenvironment could therefore serve as a platform for targeting vascular and microenvironmental interactions [109]. In another example, Liu et al. established a transwell co-culture system of mouse and human PLC-derived organoids with cancer-associated fibroblasts (CAFs) in vitro. CAFs promote organoid growth through paracrine signaling, and cancer cells in turn influence the production of secretory factors by CAFs. Furthermore, they studied the effects of CAFs on organoids derived from in vivo tumors and found that compared with transplanting organoids alone, the co-transplantation of organoids and CAFs had a higher success rate of tumor formation and heavier tumor weight. Even in the group of mice transplanted with organoids alone, the generated tumors could effectively recruit CAFs. The study indicates that the co-transplantation of CAFs and liver tumor organoids promotes tumor formation and growth in xenograft models. This model investigated the interactions between these two cell types and demonstrated the clinical significance of CAFs as important components of the TME in liver cancer development and drug resistance [110].

#### Novel markers

Numerous studies indicate that tumor lineages are linked to biomarkers to some extent. Tumor markers, which include proteins, carbohydrates, and nucleic acids, can be detected in patients’ blood, urine, and tissue samples. Furthermore, biomarkers have been validated as relatively simple and intuitive methods for the diagnosis, prognosis, and therapeutic assessment of PLC in conjunction with imaging [111]. AFP [112] and glycoconjugate antigen 199 (CA199) [113] are important indicators for the diagnosis of HCC and ICC. Elevated levels of these tumor markers are associated with high tumor incidence, low survival, and poor late prognosis in patients. These markers are also associated with microvascular tumor infiltration and invasiveness of tumor dedifferentiation [114].

However, the limitations of marker specificity and monitoring sensitivity prevent effective screening for PLC, precluding the use of these markers in some cases. For example, a retrospective analysis of liver transplant recipients showed that approximately 30% of the patients with HCC and significant symptoms, maintained AFP levels within the normal range [115]. In another report on patients with chronic liver disease, AFP detection alone was not recommended as a diagnostic marker. If the detection threshold is set low, it may lead to a high proportion of false positives, increasing the psychological pressure of patients and medical costs. If the optimal cutoff value of AFP is increased to 200 ng/mL, although the patient specificity can reach more than 99%, about 80% of HCC patients will be missed, resulting in false negative results [116]. Therefore, in addition to AFP and CA199, a variety of biomarkers have been proposed for individual or combined assessment. These include molecular and biochemical cellular markers (bone bridging proteins, de-γ-carboxy prothrombinogen, and alpha-fetoprotein-L3) [117], cancer stem cell markers (CD44, CD133, CD90, and EpCAM) [118], and non-cellular components (transforming growth factor beta, FGF, and vascular endothelial growth factor) [119]. These potential biomarkers should be further investigated in a tumor-organoid environment.

Presently, other notable markers have been identified. For example, Broutier et al. used RNAseq to analyze differentially expressed genes. They identified markers associated with HCC (chromosome 19 open reading frame 48, ubiquitin conjugating enzyme E2 S, and deoxythymidylate kinase) and ICC (complement C1q binding protein and stathmin 1). Kong et al. analyzed microarrays of six liver tumor tissues and discovered that the expression of the transmembrane tight junction protein claudin 6 (CLDN6) progressively increased from normal to paraneoplastic to HCC tumors. Furthermore, CLDN6 expression was significantly correlated with the poor survival of HCC patients, which was clinically significant. The high expression of CLDN6 promoted the proliferation of HCC cell lines; this phenomenon was also verified in organoid cultures, wherein CLDN6 increased the ability of organoid sphere formation and the formation of organoids of greater size and number [120]. Finally, Zou et al. discovered that CD39 induces apoptosis, enriches reactive immune cells, and serves as a prognostic marker for immunotherapy [121].

Similarly, novel markers have provided new ideas for liver cancer treatment, with marker aptamers carrying anticancer drugs, such as the broad-spectrum drug Adriamycin, which can be used as a targeted, safe, and controlled therapeutic option. Nucleic acid aptamers are chain-breaking structures with low immunogenicity and high tissue penetration that can be attached to the target by a variety of physical binding forces. For example, Zhou et al. tagged nucleic acid aptamers to link EPCAM, a surface marker that is overexpressed in HCC compared to normal hepatocytes, to adriamycin (EpCAM-apt-Dox). The drug-carrying complex can rapidly enter and diffuse into HCC cell lines (Hep3B and Huh7) and can increase the accumulation and retention time of DOX in cells. Hep3B cells treated with EpCAM-apt-Dox formed smaller and fewer tumor spheres than the control group. In addition, the downregulation of stem cell surface markers (AFP, EpCAM, CD133, CD24, and ALDH1A), Wnt and Notch pathway genes, and drug resistance genes was observed, indicating that the EpCAM-apt-Dox targets and impairs cancer stem like cell properties. Organoids were further established with surgically obtained liver tissues/tumors. EpCAM-apt-Dox treatment can inhibit the viability of HCC organoids, but the cytotoxicity of normal liver organoids is low. In vivo antitumor models in mice also suggest that EpCAM is an ideal target for HCC therapy with targeted cytotoxicity against tumors. This study provides a useful strategy for targeted treatment of liver cancer, and EpCAM-apt can be used as an effective drug delivery vehicle [122]. Similar experimental results were obtained for CD133 and adriamycin (CD133-apt-Dox) [123].

### Drug screening and evaluation

In terms of drug treatment, systemic antitumor therapy is currently the clinical choice, including molecularly targeted drugs, immune checkpoint inhibitors, chemotherapy, and hepatoprotective as well as choleretic-supportive therapies [124]. First-line antitumor drugs include sorafenib, lenvatinib, and atilizumab combined with bevacizumab. However, therapeutic agents for liver cancer are still lacking at this stage, as evidenced by the limitations in number and type. Therefore, a model that can rapidly validate drug activity while preserving the epigenetic and phenotypic characteristics of patients with liver cancer will help advance liver cancer treatment.

Tumor organoids can be used as drug response reactors, as they maintain cell polarization and cell-cell interactions, for screening potential anticancer compounds or assessing the drug response and efficacy of existing clinical therapeutics. Drug efficacy is generally expressed in terms of cell viability, growth curves, area under the dose curve, and half-inhibitory concentration (IC_50_) [19, 125]. Drug-sensitive organoids show growth inhibition, as evidenced by a reduction in their number and diameter, an effect that is often corroborated by clinical trials. Heterogeneity occurs in each organ class due to the preservation of the patient genetic mutation spectrum. For example, Xian et al. treated patient-derived PLC organoids with different concentrations of sorafenib, regorafenib, levatinib, and the mTOR inhibitor combinations RAD001/TAK228/phenformin (RTP). The treatment with sorafenib and regorafenib inhibited the growth of HCC organoids, but no significant difference was observed between the ICC and cHCC-ICC types. The RTP exhibited lower IC_50_ values, suggesting a stronger antitumor effect. The same inhibitory effect was observed in RTP-treated ICC and HCC organoids. Interestingly, RTP effectively inhibited the growth of sorafenib-resistant organoids. In addition to patient-derived organoids, organoids established from mouse liver cancer tissues showed similar results. For treating primary tumor-derived and PDX tumor-established organoids, administration of the two targeted therapeutic agents sorafenib and regorafenib inhibited organoid growth, with some differences [82].

Organoids have similar structure and function to real organs, which can provide detailed information on drug action on specific cells and tissues and help to understand the mechanisms of drug action. Further, organoids can process numerous samples in high-throughput screening, effectively improve the efficiency of drug screening, and accelerate the discovery of potential drugs. Most clinical trials involve cell lines and animal models of drug candidates; however, the success rate of phase I-III clinical trials of antitumor drugs is only 3.4% [126]. The main reason for this issue is primarily the 2D cultures, which have planar structures and do not respond to hepatotoxicity. Although animal models can simulate human physiological conditions to a certain extent, uncertain variables still lead to the poor accuracy of clinical predictions. In contrast, 3D structures are highly complex and can simulate the diffusion of drugs in tissues, which better reflects the reality of drug action. Furthermore, as the liver is the main organ of drug metabolism, testing at the organoid level facilitates a better understand of the pharmacokinetics of a given drug, yielding experimental results that are more representative of the absorption, distribution, metabolism, and excretion processes as well as toxicity of drugs [127, 128]. For example, after large-scale, high-throughput drug screening of liver cancer organoids, the main drugs and compounds identified to be potentially beneficial to patients were HDAC inhibitors (romidepsin and panobinostat), proteasome inhibitors (ixazomib, bortezomib, carfilzomib, and omacetaxel), DNA Top2 inhibitors (idarubicin, zorubicin, and tolteroderma), and inhibitors of RNA synthesis (procamycin) [39]. By further analyzing the sensitivity of PLC organoids to drugs, these drugs could be classified into four categories, the first of which was available and selectable drugs, such as ometazidine and pabilostat. The second category included drugs that displayed functional heterogeneity such as zorubicin and adriamycin. The third category included drugs with interpatient heterogeneity and could therefore only work in a subset of patients. The fourth class included ineffective drugs. The first and second category compounds showed promising preclinical activity, and their rational drug combinations and validation in organoid models provide new ideas for PLC therapy.

### Clinical research

The specific pathological type, disease stage, and genotype characteristics of different patients vary. In addition, the patient’s overall health, genetic background and living habits are also variable. This results in different treatment outcomes, complication risks, treatment responses, and prognosis for the same cancer type. Therefore, individualization of cancer treatment has become an important trend of modern medicine to ensure that patients can obtain the best treatment effect and quality of life. Organoid-guided tumor precision therapy proposes that in vitro organoid culture can be performed with informed consent from patients who failed to respond to standard treatments [129]. A high degree of consistency is observed between different organoids derived from the same patient. Most importantly, organoids can accurately predict the clinical response of most patients, reducing unnecessary adverse reactions in patients while avoiding time and resource consumption and providing a reference for the next step of treatment. The use of organoid models is also recommended to guide medication in patients with rare or refractory tumors that lack therapeutic standards.

**Fig. 4:**
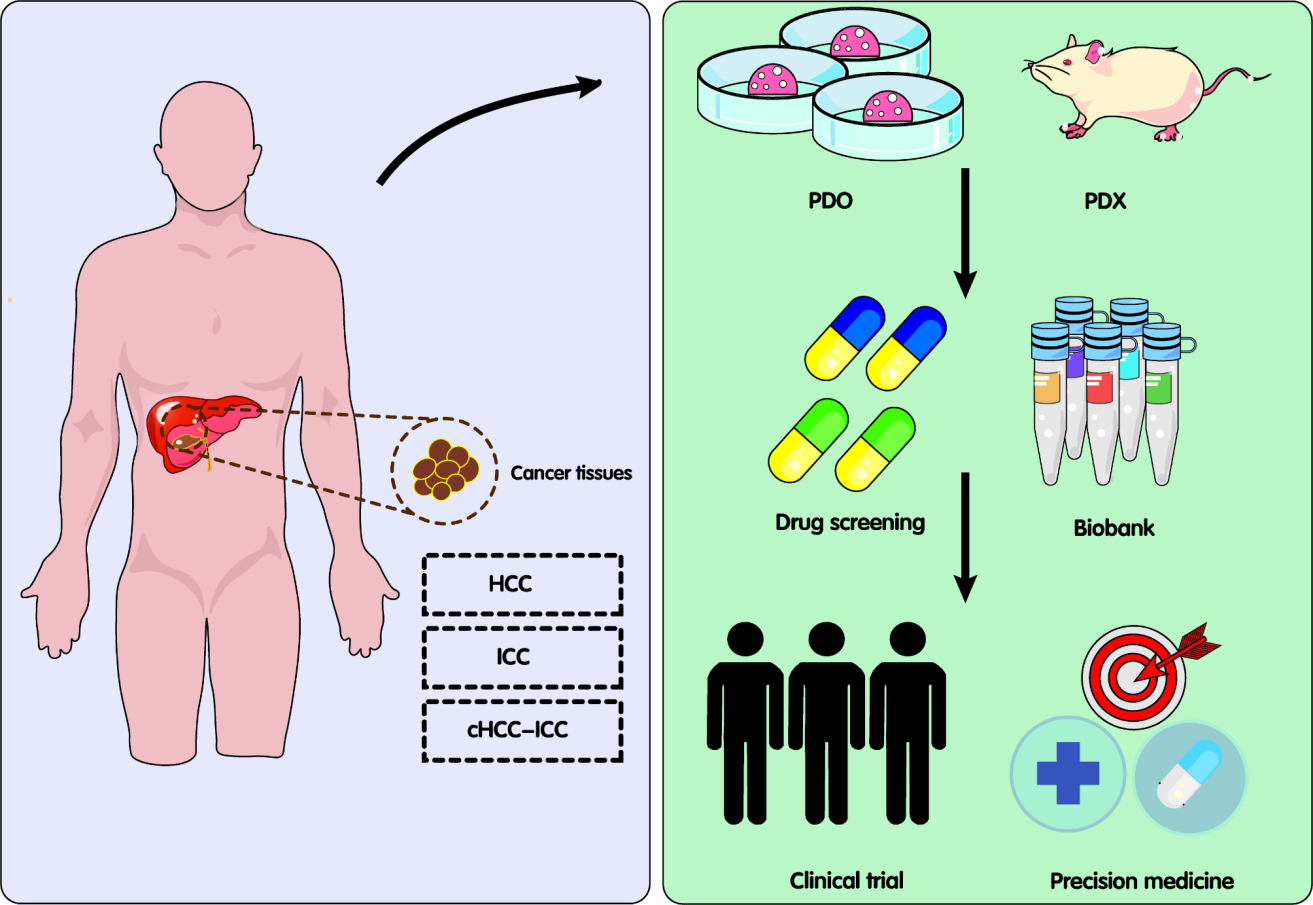
Organoids and their potential in precision medicine. Patient-derived organoids or xenografts can be used for the preclinical validation of drugs and establishment of high-quality tissue biobanks. cHCC-ICC, combined hepatocellular carcinoma-intrahepatic cholangiocarcinoma; HCC, hepatocellular carcinoma; ICC, intrahepatic cholangiocarcinoma; PDO, patient-derived organoid. PDX, patient-derived xenograft

A search of Clinicaltrials.gov (https://clinicaltrials.gov/) for the terms “liver cancer” and “organoid” shows that there are currently 12 clinical trials registrations. These include the use of liver cancer organoids for preclinical validation of drugs, in vitro culture of circulating tumor cells, establishment of high-quality tissue biobanks, and tumor metastasis prediction (Fig. 4). Details regarding some of these trials are provided in Table 2. Most of these trials are now in the subject recruitment phase. At the same time, the researchers also briefly described the possible primary and secondary research outcomes of the clinical trial according to the research purpose. Specifically, the primary measurement outcomes of organoids include: the size and quantity of organoids, the success rate of culture, genomics, and drug sensitivity results. The primary measurement outcomes of patients included: overall survival, progression free survival, objective response rate, and clinical benefit response. These clinical trials are promising to provide functional precision medicine guidance for patients with liver tumors.

**Table 2:** Clinical transformation of patient-derived liver cancer organoids

First Submitted	Identifier	Status	Enrollment	Title	Trial Aim	Biospecimen	Primary Outcome Measures
2023/10/5	NCT06077591	Not Yet Recruiting	40	Prospective Clinical Validation of Next Generation Sequencing (NGS) and Patient-Derived Tumor Organoids (PDO) Guided Therapy in Patients With Advanced/ Inoperable Solid Tumors	Evaluating the efficacy of NGS/PDO-guided treatment in patients with inoperable or metastatic solid tumors.	1.Hepatocellular carcinoma 2. Colorectal cancer	1. Tumor response, partial or complete (> 30% reduction in tumor size). [Time Frame: 6 months]
2023/6/27	NCT05932836	Recruiting	165	An Organoid-on-chips Technique Based on Biopsy Samples and Its Efficacy in Predicting the Response to HAI in HCC	Establishing an organoid-on-chips technological system based on biopsy samples and evaluating its efficacy in predicting the response to mFOLFOX6 infusion in patients with HCC.	1. Breast cancer 2. Lung cancer 3. Liver cancer 4. Bile duct cancer 5. Pancreatic cancer	1. Success rate of organoid culture of biopsy samples. The number of successful cases of organoid cultures is divided by the number of enrolled cases. [Time Frame: 2 years] 2. Accuracy of Organoid drug sensitivity test in predicting the response to mFOLFOX6 infusion in HCC patients with successful organoid culture. The sensitivity and specificity of drug sensitivity test in patients with successful organoid culture to predict the response (mRECIST) to mFOLFOX6 infusion in HCC. [Time Frame: 3 years]
2023/6/13	NCT05913141	Recruiting	30	PDO/PDO-TIL/PDOTS for Drug Screen	Using the patient-derived organoid (PDO), Patient-derived organoids-tumor-infiltrating lymphocyte co-culture (PDO-TIL), and patient-derived organotypic tissue spheroids (PDOTS) systems to simulate the tumor microenvironment in patients with cancer.	1. Liver cancer 2. Metastatic liver cancer	1. Objective response rate (ORR) evaluated by researchers based on the RECIST 1.1 standard. [Time Frame: 1 year]
2022/11/30	NCT05644743	Not Yet Recruiting	40	Clinical Transformation of Organoid Model to Predict the Efficacy of GC in the Treatment of Intrahepatic Cholangiocarcinoma	First, relevant organoids were constructed from puncture samples of unresectable ICC patients. Second, based on the intrahepatic cholangiocarcinoma organoid model, the clinical efficacy of GC regimens was predicted and in vitro and in vivo drug screening was performed to explore the significance of patient-derived tumor organoids in guiding clinical treatment. Then, the multi-omics data of organ tissues and in vitro and ex vivo drug efficacy evaluation models were used to explore the drug resistance genes of intrahepatic cholangiocarcinoma, which provided the basis for individualized drug screening and efficacy evaluation of intrahepatic cholangiocarcinoma.	1. Intrahepatic cholangiocarcinoma	1. Consistency of organoids and clinical patient responses to drugs. A total of 20 unresectable ICC patients were selected, and biopsies were performed before treatment to construct organoid models. All patients were treated with GC chemotherapy. Drug responses of organoids and clinical patients were compared to determine the feasibility of in vitro organoid culture as a drug screening platform. The samples of 3 patients who were sensitive to GC and 3 patients who were resistant to GC were sequenced to search for drug-resistant genes, and the differential drug-resistant genes were studied in vitro. [Time Frame: 3 years] 2. Construction of a drug resistance prediction model. A total of 20 patients with advanced unresectable ICC were selected to verify whether they participated in drug resistance by combining 1–2 drug resistance genes previously screened and the currently recognized platinum-based drug resistance genes. The results were compared with those of organoid models to build an organoid-based drug resistance prediction model. [Time Frame: 3 years]
2022/5/17	NCT05384184	Recruiting	48	Next Generation " Pre-clinical Model for Colorectal Cancer Metastases and Hepatocellular Carcinomas (BORG)	Evaluating the feasibility of building a biobank of liver-derived organoids, from liver metastases of colorectal cancers, hepatocellular adenoma, and adenocarcinoma (waste tissues).	1. Colorectal cancer metastases 2. Hepatocellular carcinomas	1. Build the next-generation biobank of liver-derived organoids. Grow and store organoids derived from liver biopsies (HCC and CRC mets). [Time Frame: 2 years]
2022/1/24	NCT05242237	Recruiting	300	Prognostic Value of Liver Cancer CTCs Isolated by a Novel Microfluidic Platform	Exploring in vitro cultures of CTCs by organoid culture or spheroid culture to obtain CTC cell lines to reveal the mechanism of HCC metastasis.	1. Hepatocellular carcinoma 2. Circulating tumor cell	1. Time to progression or death (months) after initial diagnosis will be recorded. [Time Frame: 2 years]
2020/9/11	NCT04561453	Recruiting	20	Feasibility Study of Multi-Platform Profiling of Resected Biliary Tract Cancer	This study will test the ability to successfully obtain results from certain personalized tests for patients with biliary tract cancers that can be surgically removed.	1. Biliary tract cancer 2. Cholangiocarcinoma 3. Gallbladder cancer 4. Intrahepatic cholangiocarcinoma 5. Perihilar cholangiocarcinoma 6. Extrahepatic cholangiocarcinoma 7.Hilar cholangiocarcinoma 8. Distal bile duct cancer	1. Success rate of organoid culture and drug screening. The investigators will measure the percentage of patients for whom organoids can be successfully cultured and subjected to a drug screen. [Time Frame: Within 12 weeks after surgery] 2. Success rate of obtaining circulating tumor DNA (ctDNA) quantification and the ability to assess the change in ctDNA levels across those time points. The investigators will measure the percentage of patients for whom ctDNA was collected and quantified at multiple key time points across the course of a patient’s treatment. [Time Frame: Through study completion, an average of 4 years.]
2016/3/5	NCT02718235	Unknown Status	200	Prospective, Multicenter HCCIS Evaluation Study	Building a database with HCC tumor organoids and testing the effect of CD8 + IL-33 + effector-memory cells on HCC tumor organoids of the respective HCCIS risk groups.	1. Hepatocellular carcinoma	1. The HCC immune score (HCCIS) is a survival predictor for patients after HCC resection. Overall survival
2015/4/30	NCT02436564	Unknown Status	75	In Vitro Models of Liver and Pancreatic Cancer	The primary objective is to develop an in vitro model of cancer for laboratory study using liver, biliary, and pancreatic cancer tissues. The secondary objective is to study the genetic and cellular biology of cancer of the liver, biliary tract, and pancreas.	1. Cholangiocarcinoma 2. Hepatocellular carcinoma 3. Pancreatic neoplasm	1. Number of tumor-derived organoids successfully cultured in vitro for a minimum of 3 months. [Time Frame: 3 years]

CRC, colorectal cancer; CTC, circulating tumor cell; ctDNA, circulating tumor DNA; GC, gemcitabine and cisplatin; HAI, hepatic artery infusion; HCC, hepatocellular carcinoma; HCCIS, the HCC immune score; ICC, intrahepatic cholangiocarcinoma; ORR, objective response rate; PDO, patient-derived tumor organoids; PDO-TIL, patient-derived organoids-tumor-infiltrating lymphocyte co-culture; PDOTS, patient-derived organotypic tissue spheroids; NGS, next generation sequencing

### Limitations and prospects of liver cancer organoids

**Fig. 5:**
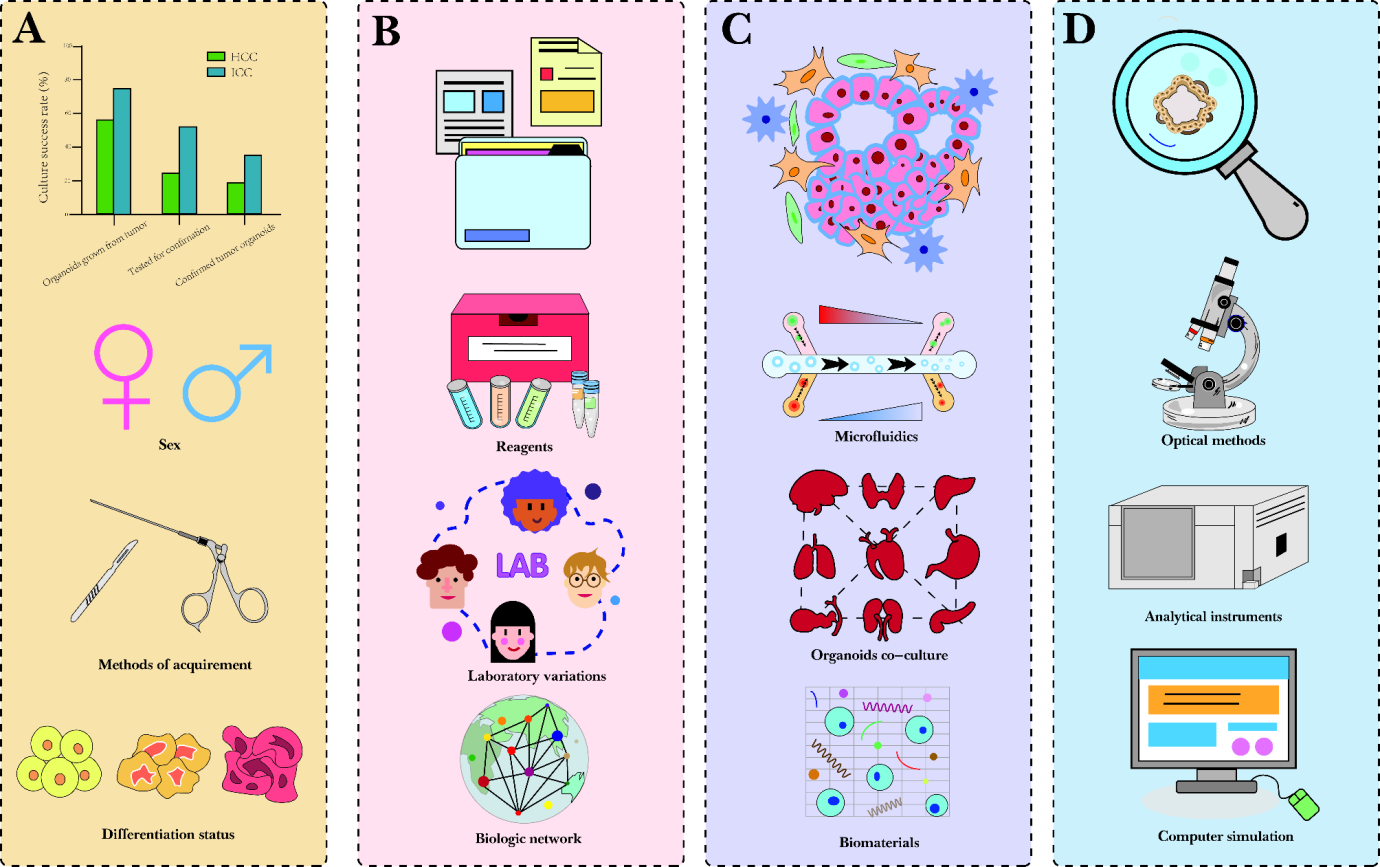
Limitations and prospects of liver cancer organoids. (**A**) Organoid establishment has a low success rate, though female patients exhibit a higher success rate of organoid modeling, indicating a dependency on the inherent proliferative capacity of primary HCC cells. (**B**) The standardization of organoid establishment is lacking. The small molecules or growth factors used in organoid culture should have defined compositions, and organoids should be characterized at different levels after a long period of culture to facilitate organoid mapping. (**C**) The tumor microenvironment and co-culture modalities in organoids are lacking. Some techniques, such as microfluidics and the use of biomaterials such as engineered ECM, can improve culture conditions and facilitate heterogeneous large-scale cultures. (**D**) Methods for organoid reading are currently limited to electron microscopy imaging and protein quantification. More computational tools and clinical validation are required. HCC, hepatocellular carcinoma; ICC, intrahepatic cholangiocarcinoma

Organoids serve as preclinical cancer models that are easy to expand and maintain while retaining heterogeneity of the original tumor, and can be manipulated at the genetic level for high-throughput drug and genome-wide screening. Compared to other models, organoids have obvious advantages that connect basic research and precision medicine. However, current organoid cultures have certain limitations [130], which are summarized in Fig. 5.

First, the success rate of liver cancer organoid modeling is low, as demonstrated in a multicenter evaluation of hepatobiliary tumor organoids that suggested that the overall establishment efficiency of PLC organoids was 28.2%, and only 19.3% for HCC. For HCC organoids, sex was the only predictor of success modeling, with female patients having a higher success rate, and the organoids were highly dependent on the inherent proliferative capacity of the patient primary HCC cells (which correlated with Ki67 level). A possible cause is the lack of epithelial stem cell characteristics required for the propagation of HCC organoids [131].

Second, a consensus among institutes on protocols regarding the establishment or nomenclature of organoids is lacking. As such, the addition of growth factors varies slightly between laboratories and the criteria for defining successful establishment vary. The culture conditions for organoids should be clarified, and small molecules or growth factors with defined compositions should be used whenever possible. Furthermore, characterizing organoids at different levels after a long period of culture is important, allowing detailed analysis of whole organoids as well as the individual cells within them. Organoid mapping can then be established in an ideal state to refine the biological network. In terms of nomenclature and classification, organoids can be subdivided into epithelial, multi-tissue, and multi-organ organoids based on their characteristics. Their names should mainly be considered for in vitro cellular models, clearly defining the cells and originating tissues [132].

Third, there are fewer organoid and TME or co-culture models, including multicellular and organoid co-cultures [133]. Engineering approaches allow precise control of the geometric input and output flow conditions, nutrient supply, and shear stress stimulation, as well as the local mechanical properties of the growing 3D tissue [134]. Microfluidic technologies such as organoids on a chip can mimic the basic functions of peristaltic contraction and vascular delivery of oxygen and nutrients while removing waste products. In organoid cultures, microfluidic systems can control organoid size and deliver analog in vivo signal gradients. Hence, large-scale culture of organoids in complex microenvironmental processes can therefore be achieved [33]. In particular, liver organoids can acquire metabolic capacity under these conditions, and some researchers have used microfluidic procedures in conjunction with organoids for drug safety and toxicity testing. Engineered ECM is another new approach for optimizing culture conditions, and the use of well-characterized engineered materials can improve adsorption and produce replicable organoids by altering mechanical regulation or covalent protein cross-linking. Engineered materials without animal components have been developed to address the shortcomings of the existing organoid support matrices. For example, organoids and infiltrating immune cells can be remodeled on artificial scaffolds to support immunotherapy experiments [135].

Finally, organoid reading methods are currently limited mainly to electron microscopy imaging and protein quantification, with an incomplete analysis of the composition of some secretions [101]. Future optimization of culture models using computational tools as well as more clinical validation is needed [136]. Follow-up studies should involve other unexplored cell types to search for more accurate systems for tissue reconstruction and multi-organ fusion.

## Conclusions

As described above, several sources of liver cancer organoids have been previously identified. Organoids serve as functional in vitro models that improve our understanding of cancer biology and pathogenesis. They also allow for the design of personalized therapeutic regimens for patients with liver cancer. In conclusion, utilizing cancer organoids is a promising strategy for advancing precision medicine.

## Data Availability

Not applicable.

## Abbreviations

2D: two-dimensional
3D: three-dimensional
AFP: alpha-feroprotein
AKT: protein kinase B
ALB: albumin
BAP1: BRCA1 associated protein 1
BME: basement membrane extract
CA199: glycoconjugate antigen 199
CAFs: cancer-associated fibroblasts
Cas: CRISPR-associated
CD133-apt-Dox: CD133 aptamer with doxorubicin
cHCC-ICC: combined hepatocellular carcinoma- intrahepatic cholangiocarcinoma
CLDN6: claudin 6
c-myc: myelocytomatosis viral oncogene homolog
CRISPR: clustered regularly interspaced short palindromic repeats
DNAJB1: DnaJ heat shock protein family (Hsp40) member B1
ECM: extracellular matrix
EFG: epidermal growth factor
EpCAM: epithelial cell adhesion molecule
EpCAM-apt-Dox: EpCAM aptamer with doxorubicin
ERK: extracellular regulated protein kinases
FGF: fibroblast growth factor
FGFR2: fibroblast growth factor receptor 2
FLC: fribolamellar carcinoma
FSTL1: follistatin-like protein 1
H&E: hematoxylin-eosin
HB: hepatoblastoma
HBV: hepatitis B virus
HCC: hepatocellular carcinoma
HCV: hepatitis C virus
hiHep: human hepatocytes
HGF: hepatocyte growth factor
HSP: heat shock protein
IC_50_: half-inhibitory concentration
ICC: intrahepatic cholangiocarcinoma
IL-8: interleukin-8
iPSCs: induced pluripotent stem cells
LICOB: liver cancer organoid biobank
MCP1: monocyte chemoattractant protein 1
MMP-9: matrix metalloproteinase 9
mTOR: mechanistic target of rapamycin
Nf2: neurofibromin2
PDO: patient-derived organoid
PDX: patient-derived xenograft
PI3K: phosphatidylinositol-3 kinase
PLC: primary liver cancer
PRKACA: protein kinase cAMP-activated catalytic subunit alpha
TACE: transcatheter arterial chemoembolization
TME: tumor microenvironment
Tsc2: tuberous sclerosis complex 2
Pten: tensin homolog
Tp53: tumor protein 53
ZO-1: zona occludens 1
